# Analysis of health economics assessment reports for pharmaceuticals in France – understanding the underlying philosophy of CEESP assessment

**DOI:** 10.1080/20016689.2017.1344088

**Published:** 2017-07-16

**Authors:** Mondher Toumi, Anastasiia Motrunich, Aurélie Millier, Cécile Rémuzat, Christos Chouaid, Bruno Falissard, Samuel Aballéa

**Affiliations:** ^a^ Faculté de Médecine, Laboratoire de Santé Publique, Aix-Marseille Université, Université de la Méditerranée, Marseille Cedex, France; ^b^ Health Economics and Outcomes Research Department, Creativ-Ceutical, Paris, France; ^c^ Faculté de médecine, INSERM U955 and Université Paris Est (UPEC)UMR U955, Créteil, France; ^d^ Département de Pneumologie et Pathologie Professionnelle, Centre Hospitalier Intercommunal DHU-ATVB, Créteil, France; ^e^ INSERM Unit U669 (Public Health and Mental Health), University Paris-Sud, Paris, France

**Keywords:** Health technology assessment, HTA, HAS, CEESP, efficiency opinion, pricing, health economics analysis, HAS guidelines

## Abstract

**Background**: Despite the guidelines for Economic and Public Health Assessment Committee (CEESP) submission having been available for nearly six years, the dossiers submitted continue to deviate from them, potentially impacting product prices.

**Objective**: to review the reports published by CEESP, analyse deviations from the guidelines, and discuss their implications for the pricing and reimbursement process.

**Study design**: CEESP reports published until January 2017 were reviewed, and deviations from the guidelines were extracted. The frequency of deviations was described by type of methodological concern (minor, important or major).

**Results**: In 19 reports, we identified 243 methodological concerns, most often concerning modelling, measurement and valuation of health states and results presentation and sensitivity analyses; nearly 63% were minor, 33% were important and 4.5% were major. All reports included minor methodological concerns, and 17 (89%) included at least one important and/or major methodological concern. Global major methodological concerns completely invalidated the analysis in seven dossiers (37%).

**Conclusion**: The CEESP submission dossiers fail to adhere to the guidelines, potentially invalidating the health economics analysis and resulting in pricing negotiations. As these negotiations tend to be unfavourable for the manufacturer, the industry should strive to improve the quality of the analyses submitted to CEESP.

## Introduction

**Figure 1.**
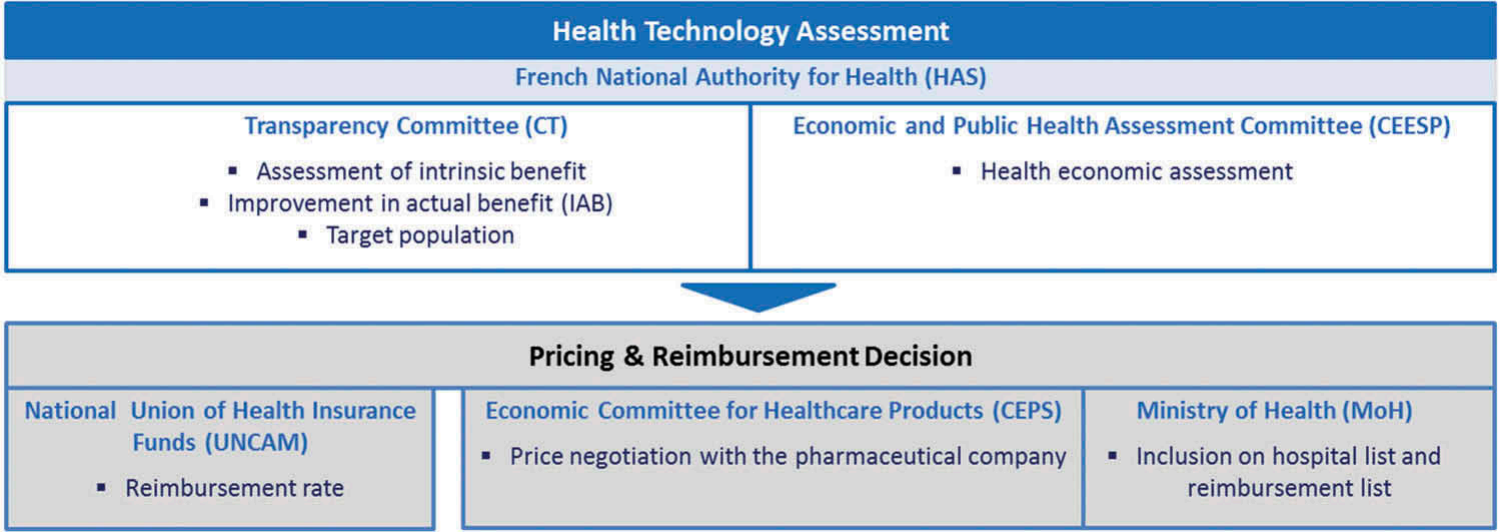
Overview of the bodies involved in the pricing and reimbursement process in France.

In France, health technology assessment (HTA) for medicines traditionally was clinically driven and was performed solely by the Transparency Committee (Commission de la Transparence, CT) – a scientific committee of the French National Authority for Health (Haute Autorité de Santé, HAS). However, in 2008, the social security funding law (LFSS) tasked HAS with assessing healthcare interventions and providing recommendations on the most cost-effective medical care and prescription strategies.[1] A dedicated commission, called Economic and Public Health Assessment Committee (Commission Evaluation Economique et de Santé Publique, CEESP), was created to fulfil this role. As such, the HTA process in France has two separate components performed by two different public bodies within the same agency – the clinical and economic assessment performed by CEESP and the public health assessment performed by CT.[2] The responsibilities of these different bodies in the pricing and reimbursement process are shown in Figure 1. CT is in charge of assessing the improvement in additional benefit (IAB) over the next best alternative and will assign a score from I to V; IAB scores of I to III acknowledge a substantial additional benefit (from major to moderate), IAB IV reflects a minor additional benefit, and IAB V means there is no additional benefit.[3] CEESP assesses the cost-effectiveness of medical treatments based on data submitted by the manufacturer, according to methodological principles for economic evaluation defined by HAS (a guideline related to methodological choices for economic assessment was issued by HAS in 2011 [4]). However, CT and CEESP are not supposed to share their assessments until these are final. This may lead to the committees independently performing the same analyses and the resulting duplication of work is considered a potential source of inefficiency.[2]

CEESP was initially created as an internal HAS working group with no legal entity, and was introduced in the Social Security Code as a specialised committee following the LFSS for 2012.[5] The decree 2012–1116 of 2 October 2012 specified health economic missions of HAS achieved through CEESP.[6] CEESP assesses innovative and high-budget impact products that meet two conditions: (1) Drugs for which IAB claimed by the company is major, important, or moderate (IAB I, II, or III indicated when filling the TC dossier; and (2) the drug is likely to have a significant impact on the health insurance budget (threshold set at €20 million annual revenue after the second year of commercialisation), through its impact on care organisation, professional practices, or patient care and – when applicable – the drug’s price.[7] Thus, in France health economics assessment is restricted to products that have shown a high additional clinical benefit (IAB I–III), and that impact health care system organisation or budget, or display a high price.[8] The restriction of health economics assessment to products with high additional value is not unique to France, and is shared with the Netherlands [9] and South Korea,[10] for example.

The objective of the CEESP assessment has been clearly defined as providing a tool to inform payers when setting prices for pharmaceuticals and devices.[2] Although it is unclear how this information is being used, some ideas have emerged from experience. Based on the framework agreement,[11] a product with IAB I–III has to undergo a health economics assessment performed by CEESP, the recommendations of which are sent to the Pricing Committee (Comité Economique des Produits de Santé, CEPS), responsible for negotiating with the manufacturer, setting the final price, and making it public once negotiations are completed. Products with IAB I–III are eligible for a price that is close to the average in the four largest EU countries (excluding France), although the CEPS may negotiate additional rebates with the manufacturer. Other products (IAB IV–V) have to undergo lengthier and generally inflexible pricing negotiations (constant cost for products with IAB IV vs comparator, discounted price for products with ASMR V vs. comparator). Thus, pricing is driven by comparative effectiveness (IAB score).[8] On the other hand, reimbursement is determined by the national health insurance (UNCAM) and is mainly driven by the intrinsic benefit of a product (also assessed by CT), irrespective of its comparative effectiveness. When deciding on reimbursement, the budget impact of the coverage decision is therefore outside the hands of the ultimate payer (UNCAM). While it is important to note that UNCAM representatives are *de facto* voting members of the Pricing Committee,[12] their impact on the final pricing decision remains nonetheless limited.

The HAS guideline for manufacturers submitting dossiers to CEESP recommends using cost-effectiveness analysis, where quality-adjusted life year (QALY) is listed as one of the options for measuring effectiveness.[4] Overtime, the QALY has imposed itself as the “gold standard” measure of effectiveness. However, CEESP does not use a specific incremental cost-effectiveness ratio (ICER) threshold; instead, its conclusions and opinions are based on the perceived adequacy of the health economics analysis supplied by the manufacturer. CEESP examines the dossier for deviations from the HAS methodological guidelines,[4] errors and methodological limitations, and judges their impact on the credibility of the health economics analysis results. Thus, each deviation from the guidelines leads to CEESP expressing methodological concerns on the credibility of the affected results. CEESP methodological concerns are scored on a three-level scale: (1) Minor methodological concern: item not in accordance with the current recommendations, but with limited or negligible impact on the conclusion. (2) Important methodological concern: item not in accordance with the current recommendations, expected to have an important impact on the conclusion (especially regarding uncertainty). (3) Major methodological concern: item does not comply with the current recommendations, invalidating part of (partial major methodological concern) or the entire (global major methodological concern) health economics study. Substantial methodological deviations from the HAS guidelines may lead to CEESP expressing a major methodological concern regarding the entire analysis (global major methodological concern), and judging the ICER to be uninterpretable. It is also noteworthy that a recent letter from the HAS president regarding the interpretation of cost-effectiveness analysis to the pricing committee (dated September 2016), stated that the ICER would be uninterpretable in case of high uncertainty in the cost-effectiveness analysis.[13]

Although the technical guidelines for economic evaluation were developed in 2011, along with other guidance for submission, in practice, manufacturers’ submission dossiers continue to deviate from the guidelines, in some cases containing deviations that are considered to have a major impact on the credibility of the results. The aim of this study was to review the assessment reports for pharmaceuticals published by CEESP, and to identify the type of deviations pointed out therein.

## Methods

The official HAS website was screened to identify CEESP pharmaceutical assessment reports published up to January 2017. An Excel table was used to extract (for each report): the name of the product, the international non-proprietary name (INN), date of report publication, overall conclusion on CEESP methodological concerns (minor, important or major), and IAB score. All CEESP reports were reviewed, and key deviations from the guidelines were identified and classified into two levels, presented in Table 1. In accordance with the HAS methodological guidance, the first level consisted of five main dimensions: study objectives, structural choices, measurement and valuation of health states and costs, modelling, results presentation and sensitivity analyses (SA). Second-level classification was established for four of the five first-level dimensions; the study objectives category was not subdivided. Furthermore, the following categories were divided into subtopics: cost, health states, transition estimates and SA. CEESP methodological concerns regarding each deviation were classed as minor, important or major. Descriptive statistics were performed to calculate the frequency of deviations by level, subtopic, and classification of methodological concern (major, important or minor).

**Table 1.** Description of the two-level classification of methodological concerns from the HAS guidelines.

Level 1	Level 2
Structural choices	Economic analysis and choice of outcome criterion
	Time horizon
	Comparison of strategies
	Perspective
Measurement and valuation of health states and costs	Costs Measurement and calculation Cost type Valuation
	Health states Measurement (methods and data) Valuation
Modelling	Transition estimates Data extrapolation Estimation method Reporting Efficacy data source
	Modelled population
	Model structure
	Programming errors
Study objective	–
Results presentation and sensitivity analyses	Sensitivity analyses Deterministic/probabilistic Scenario
	Presentation
	Validity (internal and external) and study limitations

## Results

Nineteen CEESP assessment reports for pharmaceuticals were identified on the HAS official website (Table 2). All of the reports had similar structure and included the following sections:

- objective of the study;
- consistency of the economic evaluation with HAS methodological guidelines, including cost-effectiveness analysis and optional budget impact analysis;
- CEESP conclusion;
- appendix presenting all the details about: submission objective, the product and its indication, regulatory history;
- appendix presenting in detail the CEESP critical analysis of the health economics study, and the results of the health economics study itself (ICER at the requested price and at other prices), taking into account uncertainty and SA.

**Table 2.** Methodological concerns expressed in CEESP reports.

Brand name	INN	CEESP report publication date	Total methodological concerns	Minor methodological concerns	Important methodological concerns	Major methodological concerns	Major overall methodological concerns
Adempas®	Riociguat	14/10/2014	14	6	4	4	
Botox®	Botulinum toxin	25/11/2014	10	8	2	0	
Daklinza®	Daclatasvir	03/02/2015	12	4	8	0	
Defitelio®	Defibrotide	18/02/2014	14	2	10	2	
Entyvio®	Vedolizumab	25/11/2014	11	7	4	0	
Harvoni®	Ledipasvir /sofosbuvir	26/05/2015	11	3	7	1	
Kadcyla®	Trastuzumab emtansine	11/03/2014	2	2	0	0	
Nexplanon®	Etonogestrel	15/09/2015	9	9	0	0	
Nplate®	Romiplostim	03/02/2015	6	1	5	0	
Olysio®	Simeprevir	14/10/2014	17	12	4	1	
Rotarix®	Rotavirus	22/07/2014	16	11	5	0	
Rotateq®	Rotavirus	16/09/2014	15	10	5	0	
Sovaldi®	sofosbuvir	15/04/2014	17	8	8	1	
Tecfidera®	dimethyl fumarate	24/06/2014	23	20	3	0	
Tivicay®	Dolutegravir	27/05/2014	11	10	1	0	
Viekirax® /Exviera®	Ombitasvir/ paritaprevir/ ritonavir Dasabuvir	09/06/2015	11	5	5	1	
Xolair®	Omalizumab	25/11/2014	16	9	7	0	
Xtandi®	enzalutamide	09/06/2015	12	9	2	1	
Zostavax®	Zoster vaccine	15/04/2014	16	15	1	0	

Fourteen of the submissions were for initial registration on the list of reimbursable medicines; two were for registration renewal and three for the extension of indication. All of the reports are summarised in Supplementary Table 1.

Overall 243 CEESP methodological concerns about methodological deviations from the HAS guidelines were identified (Table 3). Almost 63% (152) of them were minor methodological concerns, 33% (80) were important methodological concerns and 4.5% [10] were major methodological concerns. Seven assessment reports included at least one major methodological concern, 17 included at least one important methodological concern and all of them contained minor methodological concerns. Major methodological concerns mainly regarded SA and results presentation (55% of major methodological concerns), and the modelled population (18%). The most frequently criticised items are summarised in Table 4.

**Table 3.** The number of methodological concerns by level.

	Type of methodological concern					Type of methodological concern			
Level 1	Major	Important	Minor	Total level 1	Level 2	Major	Important	Minor	Total level 2
Structural choices	1 (4%)(9%)*	12 (46%)(15%)*	13 (50%)(9%)*	26 (11%)	Economic analysis and choice of outcome criterion	1 (50%)	0 (0%)	1 (50%)	2 (8%)
		
					Time horizon	0 (0%)	1 (50%)	1 (50%)	2 (8%)
					Comparison of strategies	0 (0%)	11 (52%)	10 (48%)	21 (81%)
					Perspective	0 (0%)	0 (0%)	1 (100%)	1 (4%)
Measurement and valuation of health states and costs	1 (1%)(9%)*	16 (21%)	58 (77%)	75 (31%)	Costs	0 (0%)	6 (14%)	36 (86%)	42 (56%)
		(20%)*	(38%)*		· Measure and calculation	0 (0%)	3 (14%)	19 (86%)	22 (52%)
					· Cost type	0 (0%)	1 (20%)	4 (80%)	5 (12%)
					· Valuation	0 (0%)	2 (13%)	13 (87%)	15 (36%)
					Health states	1 (3%)	10 (30%)	22 (67%)	33 (44%)
					· Measure (methods and data)	1 (4%)	10 (37%)	16 (59%)	27 (82%)
					· Valuation	0 (0%)	0 (0%)	6 (100%)	6 (18%)
Modelling	3 (4%)(27%)*	27 (34%)	49 (62%)	79 (33%)	Transition estimates	1 (3%)	9 (26%)	24 (71%)	34 (43%)
		(34%)*	(32%)*		· Data extrapolation	0 (0%)	4 (67%)	2 (33%)	6 (18%)
					· Estimation method	1 (8%)	0 (0%)	12 (92%)	13 (38%)
					· Reporting	0 (0%)	0 (0%)	5 (100%)	5 (15%)
					· Efficacy data source	0 (0%)	5 (50%)	5 (50%)	10 (29%)
					Modelled population	2 (20%)	2 (20%)	6 (60%)	10 (13%)
					Model structure	0 (0%)	13 (48%)	14 (52%)	31 (39%)
					Programming errors	0 (0%)	3 (75%)	1 (25%)	4 (5%)
Study objective	0 (0%)(0%)*	2 (100%)(3%)*	0 (0%)(0%)*	2 (1%)					
		
Results presentation and sensitivity analyses	6 (10%)(55%)*	23 (38%)	32 (52%)	61 (25%)	Sensitivity analyses	3 (8%)	17 (47%)	16 (44%)	36 (59%)
		(29%)*	(21%)*		· Deterministic /Probabilistic	3 (10%)	14 (45%)	14 (45%)	31 (86%)
					· Scenario	0 (0%)	3 (60%)	2 (40%)	5 (14%)
					Presentation	3 (25%)	3 (25%)	6 (50%)	12 (20%)
					Validity (internal and external) and study limitations	0 (0%)	3 (23%)	10 (77%)	13 (21%)
Total	11 (4.5%)	80 (32.9%)	152 (62.6%)	243 (100%)		11 (4.5%)	80 (32.9%)	152 (62.6%)	243 (100%)

*The indicated percentage is calculated by reservation type (minor, important and major). All other percentages are calculated by category level.

**Table 4.** Summary of the items most frequently criticised by CEESP.

Category	Most frequently reported criticisms
Health states and cost	Robustness of clinical data Extrapolation of survival curve from immature data while more mature data is available Management of adverse events in the model questionable No discussion on similarity of adverse events and their costs between the modelled strategies Consumed resources are derived from expert opinion, without the experts being documented Inconsistent utility data Validation poorly described
Modelling	Hypotheses and extrapolation choices for probability estimates are insufficiently justified Poor or unsatisfactory documentation of structural hypotheses used in the model Transition probabilities extrapolation not conservative Adverse events are not included in the model or not taken into account in utilities or cost calculations Aggregation of heterogeneous health states
Results presentation and sensitivity analyses	Incomplete scenario presented Transferability of results in clinical practice is highly uncertain Lack of sensitivity analyses on some parameters Ranges for parameters used in deterministic sensitivity analysis are not adequately justified Distributions of parameters used in probabilistic sensitivity analysis are not presented or not justified in a comprehensive manner
Structural choice	Choice of comparators used in the analysis does not correspond to comparators used in the indication Insufficient justification for the failure to take into account some of the comparators Time horizon is not appropriate with regard to the evolution of the pathology Perspective selected in the reference case analysis does not match the one recommended by HAS.

In the 19 CEESP reports analysed, we identified 75 deviations from the guidance on measurement and valuation of health states and costs, 79 modelling-related deviations, and 61 deviations linked to results presentation and SA. Costs and health states represented, respectively, 56% and 44% of deviations related to measurement and valuation. Deviations related to costs were captured in the following groups: measurement and calculation (52%), cost type (12%) and valuation of costs (36%). Amongst health state-related deviations, 82% were assigned to measurement (methods and data) and 18% to valuation of health states.

Deviations related to modelling concerned transition estimates (43%), model structure (39%), the modelled population (13%) and programming errors (5%). Deviations concerning transition estimates related to estimation methods (38%), efficacy data sources (29%), data extrapolation (18%) and reporting issues (15%).

Deviations from the guidelines on results presentation and SA concerned SA (59%), presentation of the cost-effectiveness results (20%), and internal/external validity and study limitations (21%). Deviations from guidance on SA were divided between probabilistic/deterministic analyses (86%) and scenario analyses (14%). Distinguishing between probabilistic and deterministic analyses was difficult, as CEESP frequently reports a single deviation for both of them.

Finally, 26 deviations were linked to structural choices, mainly to comparators that were inconsistent with clinical practice. Only two products deviations regarded as important were related to study objectives (both medications are indicated for the treatment of adults infected with hepatitis C).

## Discussion

### Major methodological concerns

Our attention was first drawn towards the major methodological concerns, due to their substantial impact on the manufacturer, whose product is excluded from automatic price setting by external reference pricing and becomes subject to complex, long and usually inflexible pricing negotiations, as for products with marginal or no additional benefit.

Results presentation issues, like missing efficiency frontier or wrong calculation for ICER (e.g. ICER pooled between heterogeneous populations), and inappropriately conducted SA (e.g. probabilistic analysis not run for the appropriate comparator, SA not done on all subgroups, no justification on probabilistic SA parameters) appear as the primary cause for major methodological concerns (six of 11). This is surprising, as it should be quite straightforward to address such comments either in the initial dossier, or following the CEESP review. The two weeks granted to address the CEESP comments should be sufficient to appropriately present the results, or provide a full SA package. Although it is possible that this required a major programming change not addressable in two weeks, it is likely that either the manufacturer misunderstood the requirements or deliberately decided not to address them. In any case the manufacturer has the possibility to ask for a clock stop during the technical exchange.

The modelling-related major methodological concerns, linked to estimation of transition probabilities, modelled population or inappropriate structure, represented three cases out of 11. If improperly handled in the initial submission, these types of methodological concerns may be difficult to deal with within the two-week window given to address the CEESP comments. The risk of such issues should be considered in the initial submission.

Finally, structural choices (e.g. inappropriate type of economic analysis and choice of outcome criterion) and health state valuation were reported once each as cause of major methodological concerns.

In our study sample, seven dossiers (37%) received a global major methodological concern, which is consistent with data based on 30 opinions previously published by HAS.[14] Ten out of 30 dossiers in that sample received global major methodological concerns. This means that the proportion of global major methodological concerns is not decreasing over time, as one could expect. The new reporting template for CEESP applications [15] may be helpful in reducing the number of major methodological concerns, as it is self-explanatory and comprehensive.

### Important methodological concerns

Important methodological concern may also have serious implications for the manufacturer, as an accumulation of important methodological concerns may lead to a global major methodological concern. Most frequently, important methodological concerns were related to modelling (27 out of 80), followed by results presentation and SA with 23 cases. Obviously, manufacturers should focus their efforts on defining a model structure that is able to capture all relevant outcomes and discriminate in a fair way between treatment strategies.

While comparative strategies are well identified and extensively reported in the CT dossier, they are often insufficiently documented for the CEESP dossier, and the choice of comparator(s) for the modelling exercise remains controversial. Although some comparators may not be identified because of their very low use in France, it is likely that some are not considered on purpose, to avoid evidencing some undesirable features. The consultation with CEESP, which happens to be quite open for this process, is the right forum to address those questions. From the authors’ experience, both parties – manufacturers and CEESP – carry the responsibility for an often too superficial discussion in these consultations. Moreover, these consultations usually take place quite late in the process, leaving little room for major adjustments. In any case, it remains the manufacturer’s responsibility to identify all comparators, and to justify why some are excluded from the base case analysis. Some examples of justification include: no price, very low market share, and no clinical data available. CEESP will not state at the early meeting whether a competitor should be included or not. It may advise whether the justification is acceptable, but will not give a definite answer.

### Delay in publishing the CEESP opinions

The most recent CEESP opinion, accessible on the HAS website in January 2017, is dated September 2015. Thus, less than half of the pharmaceutical opinions that CEESP has finalised and communicated to the Pricing Committee are publicly available. In fact, HAS only publishes CEESP opinions after the price negotiations are concluded. This situation is unique to France and rather unfortunate, as it prevents the stakeholders from learning from the experience and identifying CEESP expectations. It clearly suggests that the pricing process is not transparent, as third parties are unaware of the grounds on which the pricing discussions are based. On the contrary, the CT report – which is the main driver for pricing and reimbursement decisions – is made publicly available when final. In a transparent democracy, there is no reason to keep confidential a HTA assessment performed by a public agency that is expected to protect public health. This situation appears unfounded and unacceptable.

### CEESP input to the pricing committee (CEPS)

In its assessment report, CEESP provides multiple inputs to the Pricing Committee when the ICER estimate is considered free of major methodological concerns. Increasingly, CEESP does not limit itself to reporting deviations from the guidelines identified in the methodology, as was the case in the initial assessment reports. More and more, CEESP tends to put its assessment in perspective, to help payers interpret and better use its opinion. This trend is partly visible in the review of the published reports, but it is mainly obvious through the authors’ experience, when reviewing the most recent reports that are not yet publicly available. The broader perspective provided by CEESP includes a strategic interpretation, mainly of the level of uncertainty, the comparator, the feasibility of addressing the uncertainty for the manufacturer, the nature of future evidence that should be collected to address the uncertainty, the ICER estimate under alternative assumptions, and the robustness of clinical evidence. If there are no major methodological concerns, the CEPS will guarantee the average EU price, but will negotiate a rebate to make the net price cost-effective. Thus, the listed price will no longer reflect the cost for the national health insurance. This process resembles the English one, where manufacturers of products that represent substantial innovative features have the opportunity to negotiate a Patient Access Scheme [16] (a confidential discount) with the Department of Health to make their product cost-effective. The main difference between England and France is that there is no ICER threshold in France, so that CEESP has no clear-cut tool to judge the cost-effectiveness of an intervention, while there is a well-known, informal threshold in England. However, in its assessment reports, CEESP provides information on efficiency when the ICER is obviously very high. CEESP also provides a range of ICERs at different prices of the assessed drug, as its net price is unknown at the time of assessment, and the price estimate used by the company in the model is unlikely to be the final one granted by the Pricing Committee. Such information helps the Pricing Committee to consider the ICER under different conditions. CEPS has a doctrine close to that of the TLV,[17] as they apply a moving ICER threshold which has been reported to range between 50,000 and 250,000€ per QALY.[2] The CEESP assessment is starting to provide critical information for setting the net price, which is the relevant one from the manufacturer’s perspective.

### Uncertainty level

The contextualisation of the CEESP assessment is mostly centred on the uncertainty around the ICER estimate, and covers multiple aspects. There is a clear trend for the CEESP reports to be less technical than they used to be initially, and to provide more strategic input to support decision making. The following aspects of uncertainty are commonly discussed:

- The overall uncertainty around the estimate, and the direction in which the actual ICER may vary from the estimate, are the most frequently provided pieces of information.
- Subgroups excluded from the analysis by the manufacturers, which may not be covered by payers, and those where the estimated ICER may not apply.
- Uncertainty around the background risk estimation and how it may impact the reported ICER.
- Uncertainty related to SA, especially probabilistic analysis, remains the major point for CEESP and the primary reason for expressing a major methodological concern.
- Uncertainty related to scenarios.

### Feasibility of addressing the uncertainty

The feasibility of addressing the uncertainty has now become an explicit part of the assessment. If it is a priori impossible to address a critical aspect of uncertainty that should legitimately lead to major methodological concern, the CEESP may not express such major methodological concern.[17] This way, CEESP acknowledges the effort of the manufacturer and the lack of solution. The fact that the classification of a methodological concern depends on whether it would have been feasible to address it at the time of the submission may be a difference between HAS and NICE. Although this may be seen as fair from the manufacturer’s perspective, it does not help setting the price, as ultimately the ICER is highly uncertain and therefore not very informative for the Pricing Committee. This situation suggests that for the health economics dossier, the manufacturer has an obligation to make best efforts to conduct an appropriate analysis, but not to produce results – much unlike the regulatory submissions. If regulators cannot properly assess the benefit–risk ratio, they will not grant a marketing authorisation, independent of the efforts of the manufacturer. In contrast, if CEESP recognises that the manufacturer has made appropriate efforts to provide a suitable analysis, they will adopt a more lenient attitude.

### Modelling survival in oncology – a major source of growing uncertainty

Survival extrapolation in oncology has always been a controversial topic linked to high uncertainty. Recent immuno-oncology products (IOs), with a potential for curing a substantial proportion of patients, raised difficulties when modelling the survival curve while the data are still relatively immature. Evidence has accumulated to support that standard modelling of survival, in terms of drug effect extrapolation beyond RCT duration, does not apply to IOs. Based on the authors’ experience, CEESP remains resistant to any new modelling methods that would account for a proportion of cured patients, while NICE has acknowledged the limitations of the current methodologies. This attitude of CEESP is driven by its preference to adopt a conservative approach and avoid taking any risk with data extrapolation, which may dramatically impact the ICER. This is likely to become a more critical issue in the near future, as multiple new indications for IOs are in development, including in earlier treatment lines, and in combination with other treatments. Because of the high anticipated benefit of those therapies, they are expected to reach the market with a single phase IIB study. This means even more uncertainty for CEESP, as survival modelling will be absolutely necessary to appreciate the value of those products and the method used will dramatically impact the value assessment. Thus, survival extrapolation beyond clinical trial results will become one of the most critical and controversial sources of uncertainty for dossiers submitted to CEESP in the future.

### Further evidence to be collected

CEESP increasingly pinpoints the need for future evidence to strengthen the ICER estimate. This evidence may be related to a better understanding of current clinical practice, associated cost and outcomes. It is often focused on how the product will be used after launch, in which population, and what will be the associated outcome.

This information is an obvious call for coverage with evidence development, framing the nature of the studies to be requested by the Pricing Committee for the future reassessment of the drug. It may also trigger an earlier reassessment to address some of the uncertainties before the usual five-year reassessment period. CEESP considers that if an uncertainty may be addressed through a post-launch commitment within less than three years, it is acceptable to go for coverage with evidence development.[18] If it would require a longer period, CEESP recommends to avoid coverage with evidence development and to integrate the uncertainty in the pricing negotiation.

### ICER estimate under alternative assumptions

CEESP will, under some circumstances, run the model using different assumptions or different inputs than the manufacturer and provide this information to the CEPS. This information may be very useful for the Pricing Committee, as it may enhance the robustness of the ICER estimate and reduce the uncertainty.

### Robustness of clinical evidence

CEESP may sometimes evaluate the quality of the clinical evidence, allowing the Pricing Committee to integrate those limitations in the price negotiation with the manufacturer. Low quality of clinical evidence is attributable to the development plan and clinical trial design, which is a responsibility of the manufacturer. Therefore, the more lenient approach expected when an aspect of uncertainty is impossible to address may not apply, and the Pricing Committee may adopt a stricter attitude in the negotiation.

While the review of clinical evidence is performed by CT at HAS, it appears from recent research that the concerns of CEESP regarding clinical trial robustness are generally consistent with the CT ones. However, the CT evaluation is more comprehensive and may be, in the future, a useful source of information for CEESP.

### Transferability, generalisability and external validation

While jurisdiction transferability is rarely addressed by CEESP, and more often by CT, generalisability is consistently discussed. Unfortunately, it is not addressed in a systematic way, but rather considered through value judgement and questioning the generalisability of outcomes, with no clear assessment of how poor or no generalisability impacts the interpretation of clinical trials results. In most cases, CEESP reports substantial doubts on the generalisability of the modelling outcome, but, occasionally, they may instead state that the generalisability is high. The top-line level of assessment makes it very difficult to integrate such non-quantitative judgement into the pricing negotiations. This is clearly an area for improvement in the future.

External validity remains an important question and needs to be well addressed by the applicant, as it may jeopardise the pricing outcome. If the external validity is not well addressed in the manufacturer’s application, it will lead to an overall poor CEESP assessment and raise questions regarding uncertainty. Thus, the applicant should make substantial efforts to address external validity issues in the health economics analysis, for example through confronting the model with epidemiological evidence, burden of illness studies, and other models.

### Internal validation

Internal validation is an important point for the CEESP assessment. Whatever the quality control performed, any model – especially when complex – may never be completely free of errors. Of the 19 models analysed, four programming errors in three different models were reported by CEESP. Out of the four, three led to important methodological concerns. If the applicant has documented an extensive internal validation, then the discovery of programming errors would be less detrimental to the outcome of the assessment.

### Reassessment

In the reassessment of a product, which is expected every five years in France, providing national real-world evidence to feed the model is highly appreciated by CEESP. In contrast, not providing such information raises questions regarding the good will and commitment of the manufacturer, and may lead to a more stringent assessment. This real-world evidence may be based on database analysis, registries, ad-hoc cohort studies or chart abstraction. Any efforts to use national data to evaluate the efficiency of a product in the French context is seen as positive by CEESP.

### Condition for efficiencies

CEESP is expected to state the conditions for efficiency of a new product, which are considered important for price negotiation by the Pricing Committee. However, this concept is unclear, and no definition is yet available. In the framework agreement (accord cadre) between the Pricing Committee and the French National Research Based Pharmaceutical Union (LEEM),[19] the conditions for efficiency are referred to as a criterion, with no definition, and it is explicitly stated that CEESP will inform the manufacturer about this concept and how to address it. This results in a strange situation, where the manufacturer has to sign a binding framework, in which an element critical for the price negotiation is neither defined nor explained, and will be clarified after the signature. It is expected that a product is efficient when the ICER is below a given threshold, so the conditions for efficiency should be the circumstances (subpopulation, price, condition of use, etc.) that result in an ICER below that threshold. In the absence of a firm threshold, the condition of efficiency is expected to address the circumstances under which the product’s efficiency is improved. As such, it can only provide relative information, on whether some conditions may be more efficient than others.

Initially, the CEESP opinion was supposed to provide an assessment of the deviations from the HAS guidelines within the methodology of the submitted dossier. This is now the first step of the CEESP conclusion. The second step considers the conditions for efficiency, reflecting a growing role of CEESP in advising the Pricing Committee, but will be documented only if no global major methodological concern has been expressed. During this second step of assessment, CEESP will assess the conditions for efficiency and interpret the efficiency frontier. The efficiency frontier is, however, not an appropriate way to overcome the lack of ICER threshold that allows drawing conclusions on efficiency.[20,21]

### Inefficiencies to be addressed

The French health economics assessment remains inefficient in some aspects. There is a duplication of work between CEESP and CT, as CEESP does not benefit from CT input. This duplication of work within the same public organisation is unlikely to persist, as there is a high requirement for improving the efficiency of governmental administration.

It is considered that about 50% of CEESP opinions will not be used, as – ultimately – after the CEESP assessment, CT may not grant the IAB score of I–III that makes the product eligible for health economics assessment.However, it is possible that the Pricing Committee might make use of these assessments during price negotiation.

The lack of clarity about a threshold that *de facto* implicitly exists, renders CEESP conclusions complex to formulate and often unclear. CEESP will accommodate the issue in its wording and perform multiple interpretations of the results for different potential thresholds. Moving towards an explicit threshold is now imperative for producing clear and easily interpretable opinions.

## Conclusion

Overall, the importance of health economics for the pricing of innovative pharmaceuticals has grown within the French environment. CEESP has gained high credibility through professional and expert management of its duties defined by law. The quality of the CEESP assessment reports is recognised and the intellectual acuity of the assessors is obvious when reviewing them. Over time, the level of CEESP expectations has been increasing, and applications should not be expected to differ in terms of strength and quality from those submitted to NICE. The influence of the CEESP assessment on the Pricing Committee decisions is growing fast. For the manufacturers, the consequences of global major methodological concerns are severe, warranting continuous efforts to match the CEESP expectations. At more than a third, the proportion of dossiers submitted to CEESP that warrant a global major methodological concern is currently too high and could be expected to decrease over time, thanks to the growing experience of the applicants, the development of study reporting templates, and further specification of the HAS health economics guidelines. The most frequent major methodological concerns, related to results presentation and SA, should be better addressed by applicants in the future. The applicants’ efforts to file an appropriate dossier will substantially increase to match the efforts made when preparing NICE dossiers in the UK. The French pharmaceutical market is by far larger than the UK one,[22] and has shown to be friendlier for innovative medicines. Therefore, pharmaceutical companies willing to achieve their target price and revenue in the market that is the third largest globally and the largest in the EU (the same size as Germany) [22] will have to ensure they have compelling and robust health economics evidence. However, the delay in publishing CEESP opinions, the lack of clear recommendations due to the absence of an ICER threshold, and reporting in French significantly diminish the international impact of CEESP, especially when compared with NICE, SMC or TLV. The aforementioned inefficiencies and the lack of threshold remain an important issue for HAS and CEESP, and are likely to be addressed in the near future. Overall, the French health economics and HTA experience is successful, and will become an unavoidable step for recognising the value of innovation, and also for pricing of products with minor improvement (IAB IV). Furthermore, introducing the CEESP assessment in France has contributed to the fast development of health economics expertise of academic institutions, governmental agencies and private organisations observed in recent years.

## Supplementary Material

Supplementary_data_changed.docx

## Geological information

The study concerns France.
