# Supplementary material for: Analysis of health economics assessment reports for pharmaceuticals in France – understanding the underlying philosophy of CEESP assessment
Source: J Mark Access Health Policy. 2017 Jul 16;5(1):1344088. doi: 10.1080/20016689.2017.1344088 (PMC5533125; doi:10.1080/20016689.2017.1344088)
Supplement: Supplementary_data_changed.docx [file zjma_a_1344088_sm2235.docx]

## Supplementary data

Supplementary Table 1

| **Brand name** | **Therapeutic area** | **Model** | **BC of ICER**  **(€/QALY)** | **Range of ICER for subpopulations**  **(€/QALY)** | **Efficiency frontier included^*^** | **Acceptability curve** | **Tornado plot** | **BIM**  **included** | **ASMR** | **Overall conclusion on** methodological concerns |
| --- | --- | --- | --- | --- | --- | --- | --- | --- | --- | --- |
| Adempas^®^ | [Cardiology, Pulmonary arterial hypertension](http://www.centerwatch.com/drug-information/fda-approved-drugs/therapeutic-area/1/cardiology-vascular-diseases) | - Markov model - Cycle length: 4 months | 239,145 | No | No | No | Yes | No | ASMR IV, V | major |
| Botox^®^ | Genitourinary, Muscle relaxant | - Markov model - Cycle length: 3 months | 23,707 | No | No | Yes | Yes | No | ASMR IV | important |
| Daklinza^®^ | Infectious diseases,  Hepatitis C | - Markov model; Cycle length: 1 year | 14,660 | 14,660 –102,073 | Yes | No | Yes | No | ASMR IV | important |
| Defitelio^®^ | Haematology, Antithrombotic | - Markov model - Cycle length:   - 1 day in the acute phase   - 1 year after the first year | 33,273 | No | No | Yes | Yes | Yes | ASMR IV | major |
| Entyvio^®^ | Gastroenterology, Ulcerative colitis or Crohn's disease | - Markov model + decision tree - Cycle length: 8 weeks | 52,845 | No | No | Yes | Yes | No | ASMR IV, V | important |
| Harvoni^®^ | Infectious diseases, Hepatitis C | - Markov model - Cycle: 1 year | Not reported | 3,021 –15,019,560 | Yes | Yes | Yes | Yes | ASMR IV | major |
| Kadcyla^®^ | [Oncology, Breast cancer](http://www.centerwatch.com/drug-information/fda-approved-drugs/therapeutic-area/12/oncology) | - Partitioned survival model - Cycle length: 1 week | 191,661 | No | No | Yes | Yes | No | ASMR II | minor |
| Nexplanon^®^ | Genitourinary system, Contraceptive | - Markov model - Cycle length: 1 year | - 1,961 €/GNIE - N/A for QALY | No | Yes | Yes | Yes | No | Absent | minor |
| Nplate^®^ | Haematology, Antihaemorrhagic | - Markov model - Cycle length: 4 weeks– | Dominant | Dominant | No | No | Yes | No | ASMR II | important |
| Olysio^®^ | Infectious diseases, Hepatitis C | - Markov model + decision tree - Cycle length: 1 year | 18,127 | 16,890 – 18,127 | Yes | Yes | Yes | No | ASMR IV | major |
| Rotarix^®^ | Vaccine, rotavirus | - Markov model - Cycle length: 1 month | 24,413 | No | No | Yes | Yes | No | Absent | important |
| Rotateq^®^ | Vaccine, rotavirus | - Transmission model + decision tree | 29,797 | No | No | No | Yes | No | Absent | important |
| Sovaldi^®^ | Infectious diseases, Hepatitis C | - Markov model - Cycle length:   - 3 months for first 8 cycles   - 1 year afterwards | Not reported | 5,866 –75,518 | No | No | Yes | No | ASMR II, III | major |
| Tecfidera^®^ | Neurology, Multiple Sclerosis | - Markov model - Cycle length: 1 year | 128,724 | No | Yes | Yes | Yes | No | ASMR V | important |
| Tivicay^®^ | Infectious diseases, HIV | - Micro-simulation process - Cycle length: 1 year | 16,526 | No | No | Yes | Yes | No | ASMR III, IV | important |
| Viekirax^®^ / Exviera^®^ | Infectious diseases, Hepatitis C | - Markov model + decision tree - Cycle length: 1 year | Not reported | 10,975 –91,954 | Yes | Yes | Yes | No | ASMR IV | major |
| Xolair^®^ | Dermatology, Spontaneous chronic urticaria (asthma) | - Markov model - Cycle length: 4 weeks | 51,447 | 45,452 –67,319 | No | Yes | Yes | No | ASMR IV | important |
| Xtandi^®^ | [Oncology, Prostate cancer](http://www.centerwatch.com/drug-information/fda-approved-drugs/therapeutic-area/12/oncology) | - Markov model - Cycle length: 1 week | 26,088 | No | No | Yes | Yes | Yes | ASMR IV | major |
| Zostavax^®^ | Vaccine, herpes zoster virus | - Markov model - Cycle length: 1 month | 26,053 | No | No | Yes | Yes | No | ASMR IV | important |

ASMR: Improvement in clinical benefit (Amélioration du service médical rendu), BC: Base case scenario, BIM: Budget impact model, HIV: human immunodeficiency virus, ICER: Incremental cost-effectiveness ratio

*Note that “No” in this column means that efficiency frontier was not included in the CEESP report, without making any assumptions on whether it was included in the manufacturer’s submission.
